# Association between the non-HDL-cholesterol to HDL- cholesterol ratio and abdominal aortic aneurysm from a Chinese screening program

**DOI:** 10.1186/s12944-023-01939-4

**Published:** 2023-11-06

**Authors:** Wenhui Lin, Songyuan Luo, Wei Li, Jitao Liu, Ting Zhou, Fan Yang, Dan Zhou, Yuan Liu, Wenhui Huang, Yingqing Feng, Jianfang Luo

**Affiliations:** 1 Guangdong Cardiovascular Institute, Guangdong Provincial People’s Hospital, Guangdong Academy of Medical Sciences, Guangzhou, Guangdong China; 2 Department of Cardiology, Guangdong Provincial People’s Hospital Zhuhai Hospital (Zhuhai Golden Bay Center Hospital), Zhuhai, China; 3 Department of Emergency and Critical Care Medicine, Guangdong Provincial People’s Hospital, Guangdong Academy of Medical Sciences, Guangzhou, China; 4 Hypertension Research Laboratory, Guangdong Provincial Clinical Research Center for Cardiovascular Disease, Department of Cardiology, Guangdong Cardiovascular Institute, Guangdong Provincial People’s Hospital, Guangdong Academy of Medical Sciences, Guangzhou, Guangdong China

**Keywords:** Non-HDL-C/HDL-C ratio, Abdominal aortic aneurysm, Ultrasond screening, Chinese population

## Abstract

**Background:**

Abdominal aortic aneurysms (AAAs) can result in high mortality upon rupture but are usually undiagnosed because of the absence of symptoms in the early stage. Ultrasound screening is regarded as an impactful way to prevent the AAA-related death but cannot be performed efficiently; therefore, a target population, especially in Asia, for this procedure is lacking. Additionally, although dyslipidaemia and atherosclerosis are associated with AAA. However, it remains undetermined whether the non-high-density lipoprotein-cholesterol to high-density lipoprotein-cholesterol ratio (NHHR) is associated with AAA. Therefore, this study was aimed at examining whether NHHR is associated with AAA.

**Method:**

A total of 9559 participants who underwent AAA screening at Guangdong Provincial People’s Hospital and through screening in two communities in Dongguan, from June 2019 to June 2021 joined in this screening program. The diagnosis of AAA was confirmed by the ultrasound examination of the abdominal aorta rather than any known or suspected AAA. Clinical and laboratory data of participants were collected. The participants were separated into a normal group and an AAA group according to the abdominal aortic status. To eliminate confounding factors, a propensity score matching (PSM) approach was utilized. The independent relationship between NHHR and AAA was assessed through the utilization of multivariable logistic regression analysis. In addition, internal consistency was evaluated through subgroup analysis, which controlled for significant risk factors.

**Results:**

Of all the participants, 219 (2.29%) participants were diagnosed with AAA. A significant elevation in NHHR was identified in the AAA group when contrasted with that in the normal group (*P* < 0.001). As demonstrated by the results of the multivariable logistic regression analysis, AAA was independently associated with NHHR before (odds ratio [OR], 1.440, *P* < 0.001) and after PSM (OR, 1.515, *P* < 0.001). Significant extension was observed in the areas under the receiver operating characteristic curves (AUROCs) of NHHR compared to those of single lipid parameters before and after PSM. An accordant association between NHHR and AAA in different subgroups was demonstrated by subgroup analysis.

**Conclusion:**

In the Chinese population, there is an independent association between NHHR and AAA. NHHR might be propitious to distinguish individuals with high risk of AAA.

**Supplementary Information:**

The online version contains supplementary material available at 10.1186/s12944-023-01939-4.

## Introduction

Abdominal aortic aneurysm (AAA) is an irreversible and parlous disease carrying a mortality rate of 67–94%, and commonly, symptoms do not manifest prior to rupture [1]. Four screening programs, which were based on randomized controlled trials from 1991 to 2004, all indicated a decline in AAA-related mortality [2–5]. Ultrasound screening is regarded as an impactful way to prevent AAA-related death and AAA rupture [6]. However, studies have reported that in Western Europe and America, the prevalence of AAA has decreased to 1.3–1.7%, which influences the effectiveness and cost- effectiveness of screening [7–9]. Therefore, parameters that could identify individuals at increased risk of AAA, should be explored to enhance the AAA prevalence in the more targeted screening for AAA [10].

AAA is an aortic dilatation of over 3 cm inside the abdominal area [11]. Patients with AAAs usually suffer from atherosclerosis simultaneously, and the relevance between peripheral atherosclerosis or coronary heart disease (CAD) and AAA has been proposed in numerous studies [12–14]. According to a 7-year prospective study, atherosclerosis risk factors were strongly connected with AAA prevalence [15]. Lately, there has been an increased focus on elucidating the importance of non-traditional lipid indicators, such as non-high-density lipoprotein cholesterol (non-HDL-C) and the non-high-density lipoprotein cholesterol to high-density lipoprotein cholesterol (non-HDL-C/HDL-C) ratio (NHHR) [16, 17], in predicting atherosclerotic cardiovascular disease [18–20]. As a novel lipid parameter, NHHR, which consists of atherogenic and antiatherogenic lipid particles, has been considered as a diagnostic marker for many dyslipidemia-related diseases, for example diabetes mellitus [21–23], metabolic syndrome [18] and carotid atherosclerosis [19, 20]. However, studies appraising the association between NHHR and AAA are limited. The present study was aimed at exploring whether NHHR was associated with AAA, and increasing the prevalence for AAA in the screening.

## Methods

### Study population

**Fig. 1:**
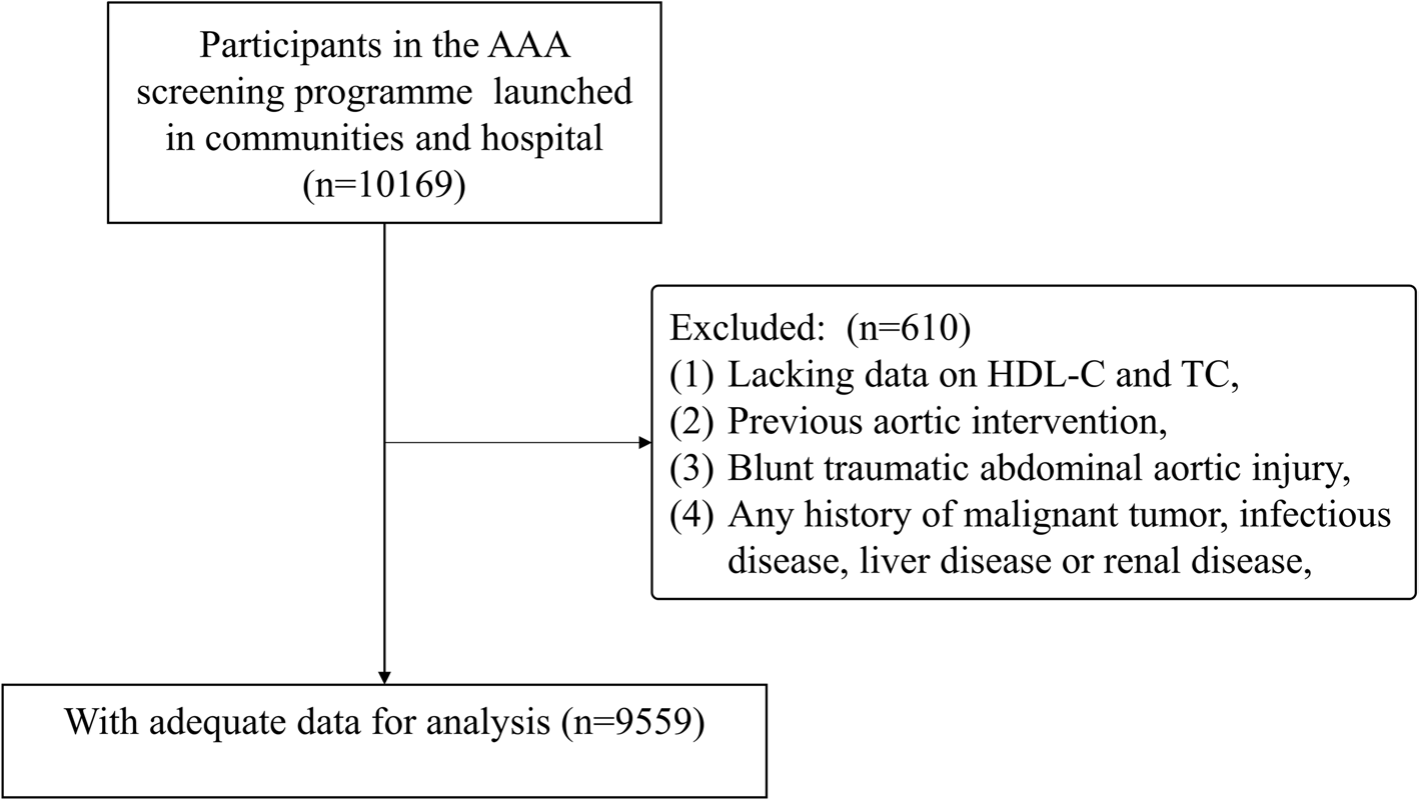
Patient selection process. AAA, abdominal aortic aneurysm; TC, total cholesterol; HDL-c, high-density lipoprotein cholesterol

The study population comprised 10,169 Chinese adults who underwent AAA screening at Guangdong Provincial People’s Hospital or through screening programs in two communities in Dongguan, China, from June 2019 to June 2021. The diagnosis of AAA was confirmed by ultrasound examination of the abdominal aorta, rather than any known or suspected AAA. In this study, the participant selection standard encompassed (1) any history of malignant tumor, infectious disease, liver disease or renal disease, (2) blunt traumatic abdominal aortic injury, (3) previous aortic intervention, and (4) a lack of data on HDL-C and total cholesterol (TC) levels. Figure 1 depicts the flowchart for the participant selection standards.

### Definition

In this study, AAA was defined as having an abdominal aortic diameter (AAD) greater than 30 mm [11]. Non-HDL-C was established through computing the numerical difference between TC (mmol/L) and HDL-C (mmol/L) [24].

### Measurement and data collection

All study participants had an ultrasound scan, which is recommended for AAA screening in the latest guidelines [25, 26]. Not only does ultrasound scanning exhibit high sensitivity (94%-100%) but it also demonstrates high specificity (98%-100%) in the detection of AAA [27–31]. Radiologists who were recruited for the screening all satisfied the undermentioned requirements: 1) Over 5 years of radiological experience should be possessed 2) abdominal aorta ultrasound scans should have been conducted at a minimum frequency of once per month over the past 12 months. Before performing measurements, radiologists must learn the measurement standards for AAA screening and adhere to these requirements. The ultrasound scans were performed in a plane perpendicular to the aortic longitudinal axis. Regarding the setting of the caliper, radiologists were required to measure the abdominal aortic diameter with an outer-to-outer (OTO) measurement, which was defined as measuring from outer anterior wall to the outer posterior wall. In addition, the measurement was started from the diaphragm, and ended at the bifurcation of the aorta. The maximal abdominal aortic diameter was defined as the largest diameter from the lowest renal artery to the aortic bifurcation in the transverse plane and the longitudinal plane [32–34].

Participants were gauged for weight and height with them donning lightweight attire and standing barefoot. The participants' information pertaining to their health, including age, smoking history, and previous medical conditions, was self-reported by the participants and documented by the researchers. Smoking was regarded as a binary variable denoting whether individuals had ever smoked (yes/no) in the past. Prior to blood sample extraction, participants were required to undergo an 8-h fasting period. Uric acid (UA), serum creatinine (Cr), low-density lipoprotein cholesterol (LDL-C), HDL-C, TC and TG were tested with the Hitachi 7600 machine (Kyowa, Japan). Conventional lipid parameters, comprising TC, TG, HDL-C, and LDL-C, could be reliably detected using enzymatic methods. HbA1c was tested with an HLC-723 G7 (Tosoh, Japan).

Demographics, clinical characteristics, laboratory and ultrasound scan findings were recorded by 2 researchers independently.

### Propensity score matching analysis

To strengthen the repeatability of the study, propensity score matching (PSM) was applied to eliminate probable confounders and selection bias of this retrospective review. The characteristics used to calculate the propensity score were age, BMI, sex, smoking, hypertension, diabetes mellitus (DM), coronary artery disease (CAD), peripheral artery disease (PAD), stroke, prior usage of angiotensin system inhibitors, beta-blockers, statins, and metformin. One to-one nearest-neighbor matching was implemented using a 0.2 caliper. After matching, 2 groups of 219 subjects were identified. Standardized mean differences (SMDs) were utilized to estimate the difference between the 2 matched groups. Commonly, it is acceptable to obtain a maximum SMD of 0.10 or even 0.15.

### Statistical analysis

Participant characteristics were considered based on the presence or absence of AAA. Continuous variables are presented, with the mean and standard deviation (SD) directed to the data that conforms to a normal distribution, and with medians along with the interquartile range (IQR) when dealing with data that does not follow a normal distribution. Student's t-test was utilized to conduct the comparisons on data demonstrating a normal distribution, while the Mann–Whitney U test was employed for data that did not adhere to normal distribution. Furthermore, in the presentation of categorical variables, they are depicted either in terms of relative frequencies (percentages). Subsequently, the comparison of categorical variables involved the application of either the chi-square test or Fisher's exact test. For the sake of estimating the association between NHHR and AAA, NHHR was categorized into tertiles [low (< 2.50 mmol/L), middle (2.50–3.51 mmol/L), high (> 3.51 mmol/L)]. An assessment of the independent association between NHHR and AAA was performed through the implementation of logistic regression analysis. As a consequence, this analysis yielded odds ratios (ORs) along with their corresponding 95% confidence intervals (CIs). Initially, univariate logistic regression analysis was conducted on all the collected variables. Multivariable logistic regression analysis was employed for investigating factors independently linked to the disease, employing variables with a *P* < 0.05 in the univariate analysis. Subsequently, three main models were constructed for adjusting the covariate, namely, Model 1, unadjusted; Model 2, adjusted for age, BMI and sex; and Model 3, adjusted for age, BMI, sex, smoking, hypertension, DM, CAD, PAD, stroke, levels of alanine aminotransferase(ALT), aspartate aminotransferase(AST), uric acid, blood urea nitrogen (BUN), Cr, TG, TC, LDL-C, HBA1C and fasting glucose, and use of angiotensin system inhibitors, beta-blockers, statins and metformin.

In the subgroup analysis, NHHR was probed to determine whether it was associated with AAA in the several subgroups, which included age, sex, smoking, hypertension, CAD and previous statin use. In every subgroup, the multiple stepwise logistic regression was implemented.

The diagnostic performance of the variables in predicting AAA was assessed utilizing receiver operating characteristic (ROC) curves. Subsequently, for the purpose of quantifying and comparing results of the analysis, the area under the curve (AUC) along with its paired 95% CI was computed. In addition, the Youden index (YI) was used to determine a cutoff value for NHHR. Based on this cutoff value, the division of participants into two groups was carried out with the objective of exploring the connection between NHHR and AAA.

## Results

### Baseline characteristics

This study comprised 9559 participants, of whom 6,144 were male (64.3%), and the average age was 70.3 ± 0.1 years. The total number of 97.7% (9340) and 2.3% (219) of the participants were distributed in the normal group and AAA group severally (details in Supplementary Table 1). Before matching, the AAA group commonly had a larger percentage of comorbidities (except for diabetes mellitus). There was no substantial difference in comorbidities between the normal and AAA groups, after matching. In the AAA group, NHHR exhibited relatively elevated values compared to the normal group, HDL-C levels demonstrated a decrease in the AAA group compared to the normal group. The baseline characteristics concerning the participants, which were separated by the abdominal aorta status, are displayed in Table 1.

**Table 1:** Baseline characteristics Stratified by the groups with and without AAA^a^

Variables	**Unmatched population**				**Matched population**			
	**Normal (*n* = 9340)**	**AAA (*n* = 219)**	**SMD**	***P***^**†**^	**Normal (*n* = 219)**	**AAA (*n* = 219)**	**SMD**	***P***^**†**^
Age (years)	70.3 ± 0.1	72.6 ± 0.4	0.345	< 0.001	72.6 ± 0.5	72.6 ± 0.4	0.004	0.946
BMI (Kg/m^2^)	23.9 ± 0.03	23.8 ± 0.2	0.043	0.507	24.0 ± 0.2	23.8 ± 0.2	0.098	0.583
Sex (male)	63.7 (5949)	89.0 (195)	0.625	< 0.001	92.2 (202)	89.0 (195)	0.110	0.251
Smoking, %	23.7 (2216)	40.2 (88)	0.358	< 0.001	37.4 (82)	40.2 (88)	0.056	0.556
Hypertension, %	54.0 (5047)	66.7 (146)	0.260	< 0.001	66.2 (145)	66.7(146)	0.010	0.919
Diabetes mellitus, %	21.6 (2013)	18.7 (41)	0.071	0.313	15.1 (33)	18.7 (41)	0.097	0.308
Coronary artery disease, %	38.8 (3627)	56.2 (3750)	0.352	< 0.001	53.0 (116)	56.2 (123)	0.064	0.502
Peripheral artery disease, %	3.3(312)	3.7(8)	0.017	0.799	5.0 (11)	3.7 (8)	0.067	0.482
Stroke, %	5.4(503)	10.0(22)	0.175	0.003	9.6 (21)	10.0 (22)	0.015	0.872
Maximal abdominal aortic diameter (mm)	19.0 (17.0–20.5)	36.0 (32.0–47.0)	2.317	< 0.001	20.0 (18.0–21.0)	36.0 (32.0–47.0)	2.303	< 0.001
ALT (U/L)	20.0 (14.7–27.7)	18.0 (14.0–25.0)	0.060	0.003	21.0 (16.0–27.4)	18.0 (14.0–25.0)	0.214	< 0.001
AST (U/L)	22.0 (18.3–28.0)	21.0 (18.7–27.5)	0.015	0.407	22.9 (19.0–28.4)	21.0 (18.7–27.5)	0.087	0.069
UA (mmol/L)	398.0 (327.9–460.0)	422.0 (368.0–504.3)	0.332	< 0.001	402.0 (351.3–483.3)	422.0 (368.0–504.3)	0.234	0.006
CR (μmol/L)	81.0 (67.0–96.8)	94.9 (77.7–118.3)	0.328	< 0.001	89.2 (75.1–105.0)	94.9 (77.7–118.3)	0.154	0.010
BUN (mmol/L)	5.5 (4.5–6.9)	6.2 (5.2–8.2)	0.320	< 0.001	5.9 (4.6–7.3)	6.2 (5.2–8.2)	0.141	0.106
TG (mmol/L)	1.4 (1.0–1.9)	1.4 (1.0–1.7)	0.087	0.912	1.4 (1.0–1.9)	1.4 (1.0–1.7)	0.039	0.245
TC (mmol/L)	4.6 (3.8–5.4)	4.6 (3.7–5.3)	0.076	0.265	4.1 (3.5–5.2)	4.6 (3.7–5.3)	0.216	0.034
LDL- C (mmol/L)	2.8 (2.2–3.5)	2.9 (2.3–3.5)	0.060	0.250	2.6 (2.0–3.3)	2.9 (2.3–3.5)	0.293	0.002
HDL-C (mmol/L)	1.1 (1.0–1.4)	1.0 (0.9–1.2)	0.102	< 0.001	1.1 (0.9–1.3)	1.0 (0.9–1.2)	0.206	0.049
non-HDL-C (mmol/L)	3.4 (2.7–4.2)	3.6 (2.8–4.2)	0.102	0.114	3.1 (2.5–3.9)	3.6 (2.8–4.2)	0.343	0.001
non-HDL-C / HDL-C ratio	2.9 (2.2–3.8)	3.5 (2.9–4.3)	0.457	< 0.001	3.0 (2.3–3.8)	3.5 (2.9–4.3)	0.449	< 0.001
Fasting glucose (mmol/L)	5.3 (4.7–6.3)	5.2 (4.5–6.6)	0.088	0.109	5.3 (4.6–6.2)	5.2 (4.5–6.6)	0.107	0.197
HBA1C (mmol/L)	6.1 (5.7–6.3)	6.0 (5.6–6.2)	0.273	< 0.001	6.1 (5.8–6.2)	6.0 (5.6–6.2)	0.236	0.021
Medication use								
Angiotensin system inhibitors, %	47.5 (4439)	56.6 (124)	0.183	0.008	54.3 (119)	56.6 (124)	0.046	0.631
Beta-blockers, %	42.1 (3932)	62.6 (137)	0.418	< 0.001	58.0 (127)	62.6 (137)	0.093	0.329
Statins, %	51.0 (4762)	67.1 (147)	0.332	< 0.001	65.8 (144)	147.0 (67.1)	0.029	0.761
Metformin, %	9.6 (895)	7.8 (17)	0.065	0.365	7.8 (17)	7.8 (17)	< 0.001	1.000

*Abbreviations*: *AAA* Abdominal aortic aneurysm, *BMI* Body mass index, *ALT* Alanine aminotransferase, *AST* aspartate aminotransferase, *UA* Uric acide, *Cr* Creatinine, *BUN* Blood urea nitrogen, *TG* Triglyceride, *TC* Total cholesterol, *LDL-C* Low-density lipoprotein cholesterol, *HDL-C* High-density lipoprotein cholesterol, *non-HDL-C* Non-high-density lipoprotein cholesterol, *non-HDL-C/HDL-C ratio* Non-high-density lipoprotein cholesterol to high-density lipoprotein cholesterol ratio, *HbA1c* Hemoglobin A1c, *SMD* Standardized mean difference

^a^Values are given as mean + standard deviation, number (percentage), or median (quartiles 1 through 3). †*P*-values were derived from Mann–Whitney U-tests for continuous variables, and Chi-square tests for categorical variables

**Fig. 2:**
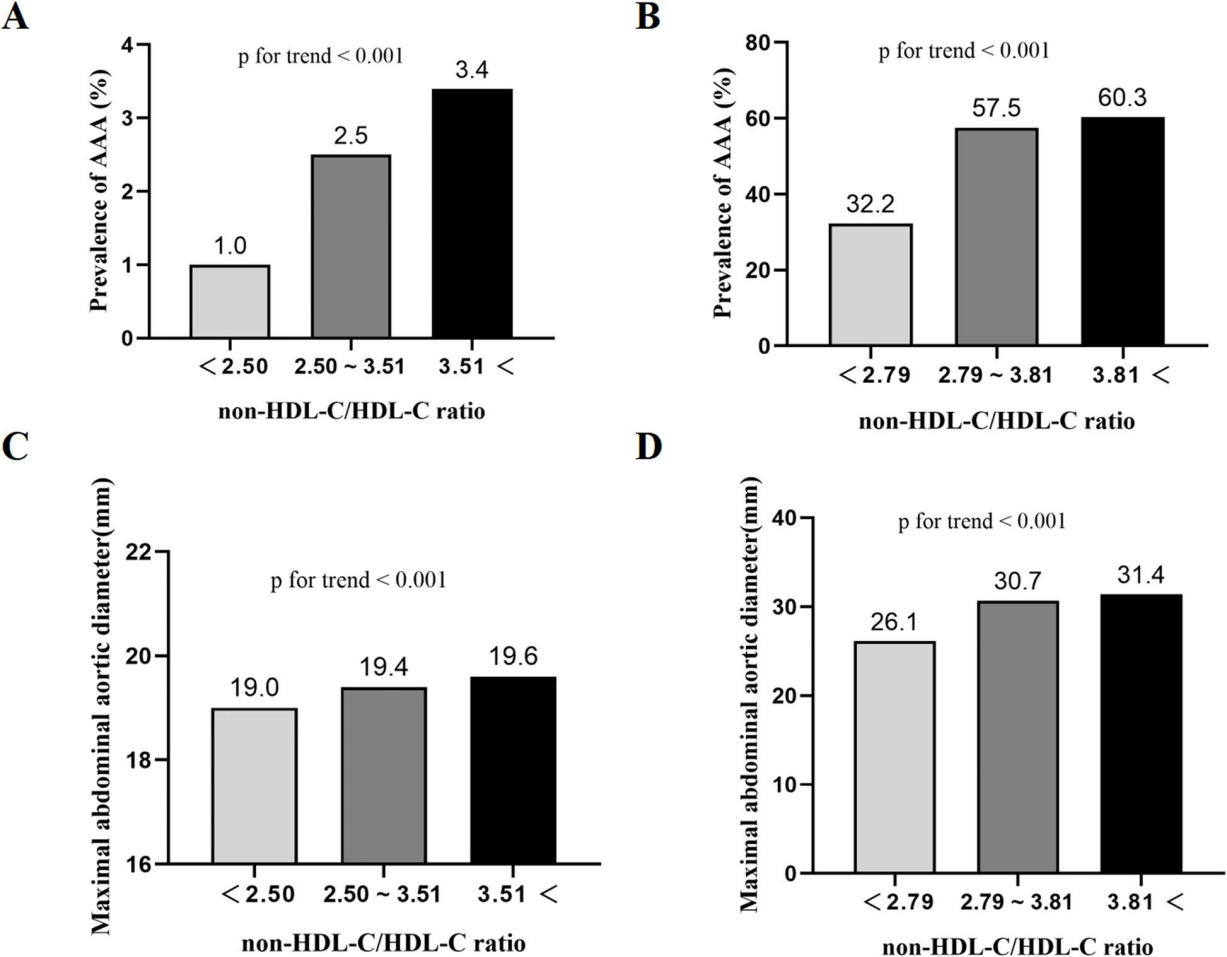
The prevalence of AAA according to non-HDL-c/HDL-c ratio tertiles before (**A**) and after PSM (**B**). Maximal abdominal aortic aneurysm according to non-HDL-c/HDL-c ratio tertiles before (**C**) and after PSM (**D**). AAA, abdominal aortic aneurysm; non-HDL-c/HDL-c ratio, non-high-density lipoprotein cholesterol to high-density lipoprotein cholesterol ratio; PSM, propensity score matching

To enhance clinical utility, three groups were formed among the participants (low NHHR group: NHHR < 2.50; medium NHHR group: 2.50 ≤ NHHR ≤ 3.51; high NHHR group: NHHR > 3.51) after dividing NHHR into tertiles. The high NHHR group exhibited a distinct increase in the prevalence of AAA in comparison to the medium NHHR group and low NHHR group (1.0% versus 2.5% versus 3.4%; *P<*0.001) (Fig. 2A). The high NHHR group showed a remarkably greater maximum AAD in contrast to both the low NHHR and medium NHHR groups. (19.0 versus 19.4 versus 19.6; *P<*0.001) (Fig. 2B).

### Univariate and multivariable logistic regression analysis

As demonstrated by the findings of univariate logistic regression analysis, NHHR exhibited a substantial association with AAA (OR, 1.391; *P* < 0.001). Other significant parameters comprised age, sex, smoking, hypertension, CAD, stroke, and levels of UA, Cr, BUN, HDL-C and HBAC1. To confirm that no multicollinearity existed among all variables, not only tolerance, but also the variance inflation factor (VIF) were assessed before conducting the multivariable logistic regression analysis with these significant factors (Supplement. Table 2). In this analysis, NHHR was still linked to the prevalence of AAA (OR, 1.440; *P* < 0.001) (Table 2). After adjusting for confounders with stepwise multivariable logistic regression analysis, when considering the low NHHR group as a reference, it was observed that the high NHHR group exhibited the strongest association with AAA. (OR, 4.231; 95% CI, (2.754–6.500); *P* < 0.001) (Table 3).

**Table 2:** Univariate and multivariable logistic regression analysis of baseline variables and prevalence of AAA in the matched and unmatched populations

	**Unmatched population**				**Matched population**			
	**Univariate**		**Multivariate**		**Univariate**		**Multivariate**	
**Variables**	**OR**	***P***	**OR**	***P***	**OR**	**P**	**OR**	***P***
Age	1.046	< 0.001	1.056 (1.035–1.077)	< 0.001	1.000	0.965		
BMI	0.986	0.544			0.963	0.304		
Sex	4.631	< 0.001	3.968 (2.555–6.162)	< 0.001	0.684	0.253		
Smoking	2.160	< 0.001	1.697 (1.264–2.279)	0.001	1.122	0.556		
Hypertension	1.701	< 0.001	1.392 (1.032–1.877)	0.030	1.021	0.919		
Diabetes mellitus	0.838	0.314			1.298	0.308		
Coronary artery disease	2.018	< 0.001			1.138	0.502		
Peripheral artery disease	1.097	0.799			0.717	0.483		
Stroke	1.962	0.003	1.515 (0.953–2.407)	0.079	1.053	0.872		
ALT	0.992	0.105			0.988	0.034	0.985 (0.974–0.997)	0.012
AST	1.000	0.881			0.998	0.394		
UA	1.003	< 0.001			1.002	0.016	1.002 (1.000–1.004)	0.018
Cr	1.003	< 0.001			1.002	0.126		
BUN	1.077	< 0.001	1.056 (1.025–1.088)	< 0.001	1.035	0.150		
TG	0.915	0.269			1.053	0.686		
TC	0.938	0.277			1.218	0.025		
LDL-c	1.062	0.392			1.404	0.003		
HDL-c	0.210	< 0.001			0.507	0.033		
non-HDL-c	1.092	0.15			1.416	< 0.001		
non-HDL-c/HDL-c ratio	1.391	< 0.001	1.440 (1.301–1.593)	< 0.001	1.520	< 0.001	1.515(1.269–1.809)	< 0.001
Fasting glucose	0.961	0.221			0.954	0.268		
HBA1C	0.733	< 0.001	0.638 (0.530–0.767)	< 0.001	0.752	0.017		
Medication use								
Angiotensin system inhibitors	1.441	0.008			1.097	0.631		
Beta-blockers	2.297	< 0.001	2.198 (1.647–2.934)	< 0.001	1.210	0.329		
Statins	1.963	< 0.001			1.063	0.761		
metformin	0.794	0.366			1.000	1.000		

*Abbreviations*: *AAA* Abdominal aortic aneurysm, *BMI* Body mass index, *ALT* Alanine aminotransferase, *AST* aspartate aminotransferase, *UA* Uric acide, *Cr* Creatinine, *BUN* Blood urea nitrogen, *TG* Triglyceride, *TC* Total cholesterol, *LDL-c* Low-density lipoprotein cholesterol, *HDL-c* High-density lipoprotein cholesterol, *non-HDL-c* non-high-density lipoprotein cholesterol, *non-HDL-c/HDL-c ratio* Non-high-density lipoprotein cholesterol to high-density lipoprotein cholesterol ratio, *HbA1c* Hemoglobin A1c

**Table 3:** Associations between the prevalence of AAA and the non-HDL-c/HDL-c ratio as continuous variables and in tertiles

**Variables**	**No. of participants (AAA patients)**	**Unadjusted OR (95%CI)**	***P* value**	**Model 1**^**a**^** OR (95%CI)**	***P* value**	**Model 2**^**b**^** OR (95%CI)**	***P* value**
non-HDL-c / HDL-c ratio	9559 (219)	1.391(1.269–1.526)	0.001	1.417(1.287–1.56)	0.001	1.584(1.411–1.778)	<0.001
non-HDL-c / HDL-c ratio							
<2.50	3187 (32)	1.000		1.000		1.000	
2.50~3.51	3186 (79)	2.507(1.658–3.791)	<0.001	2.566(1.693–3.888)	<0.001	2.633 (1.725–4.019)	<0.001
>3.51	3186 (108)	3.459(2.325–5.148)	<0.001	3.604(2.416–5.375)	<0.001	4.231 (2.754–6.500)	<0.001

^*^a Model 1 adjusted for age, body mass index and sex

^*^b Model 2 adjusted for age, body mass index, sex, smoking, hypertension, diabetes mellitus, coronary artery disease, peripheral artery disease, stroke, levels of alanine aminotransferase, aspartate aminotransferase, uric acid, blood urea nitrogen, serum creatinine, triglyceride, total cholesterol, low-density lipoprotein cholesterol, fasting glucose and hemoglobin A1c, and use of angiotensin system inhibitors, beta-blockers, statins and metformin

### Propensity score matching analysis

One-to-one nearest-neighbor matching was utilized to eliminate the possible confounding factors accordingly. Two groups, each consisting of 219 participants, were formed. After matching, NHHR in the AAA group still exceeded that in the normal group. In the meanwhile, the AAA group exhibited a reduced HDL-C level in comparison to the normal group. (Table 1). Furthermore, the high NHHR group possessed a considerably highest prevalence of AAA (32.2% versus 57.5% versus 60.3%; *P<*0.01) and maximal AAD (26.1 versus 30.7 versus 31.4; *P<*0.01) than the low NHHR group (Fig. 2C, D). Subsequently, logistic regression analyses, both univariate and multivariable, were conducted in the matched cohort. (Table 2). NHHR, which was revealed by the univariate logistic regression analysis, was in association with the prevalence of AAA (odds ratio [OR], 1.520; *P* < 0.001). Moreover, NHHR, which was proved by the multivariable logistic regression analysis, might be linked to AAA prevalence independently (OR, 1.515; *P* < 0.001).

### Subgroups analyses

**Fig. 3:**
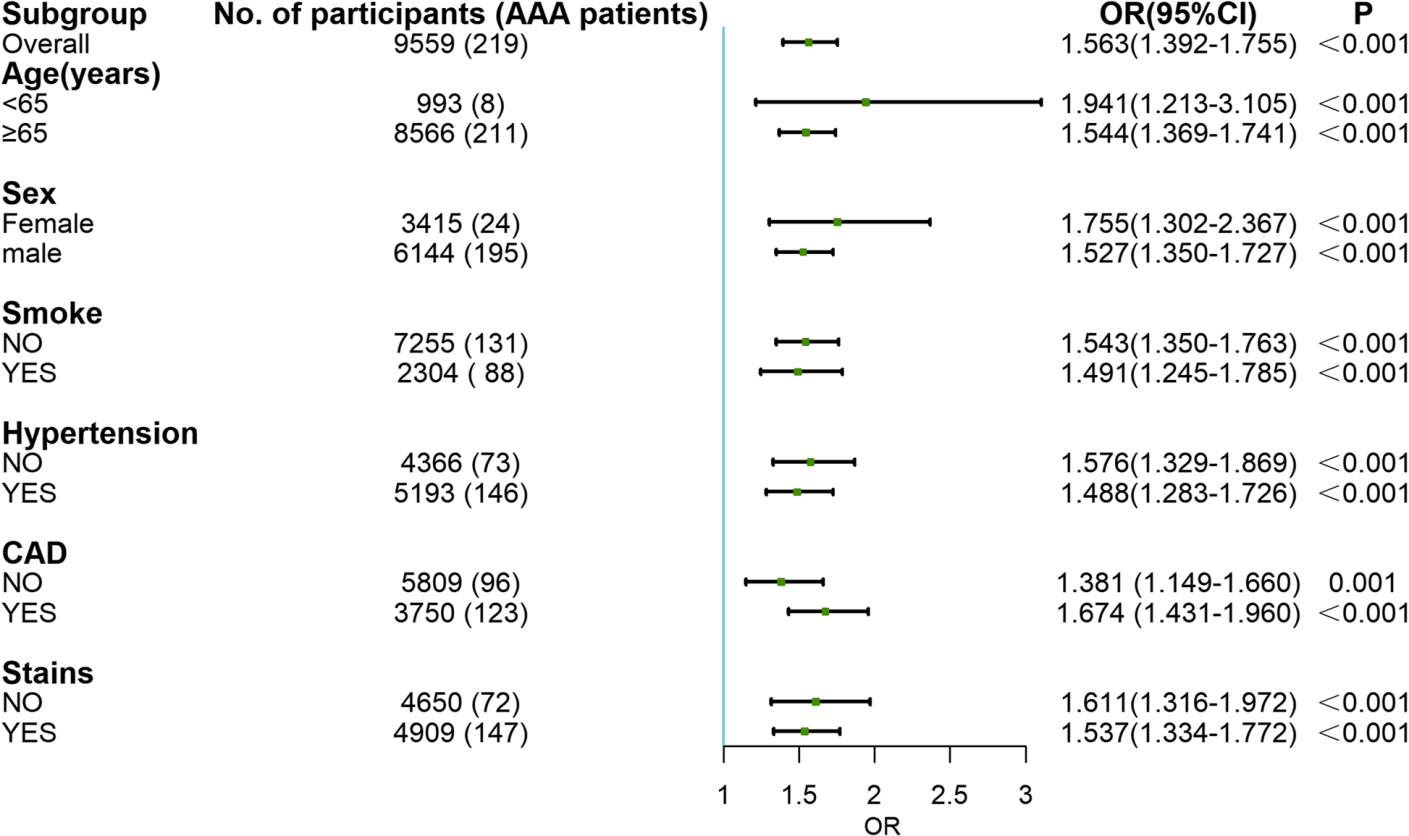
Multiple logistic regression analysis of the non-HDL-c/HDL-c ratio and risk for the prevalence of AAA in subgroups. The adjusted variables were age, BMI, sex, hypertension, diabetes mellitus, coronary artery disease, peripheral artery disease, stroke, levels of alanine aminotransferase, aspartate aminotransferase, uric acid, blood urea nitrogen, serum creatinine, triglyceride, total cholesterol, low-density lipoprotein cholesterol, fasting glucose and hemoglobin A1c, and use of angiotensin system inhibitors, beta-blockers, statins and metformin. OR, odds ratio

To validate the internal stability of the study, stratified analyses to probe the odds of AAA with changes in NHHR in different subgroups were performed. In consequence, NHHR remained substantially tied to the prevalence of AAA when considering all stratified subgroups (*P* < 0.001), which was comprised of age, sex, smoking, hypertension, CAD and previous statin use (Fig. 3).

### Receiver operating characteristic curve analysis

**Fig. 4:**
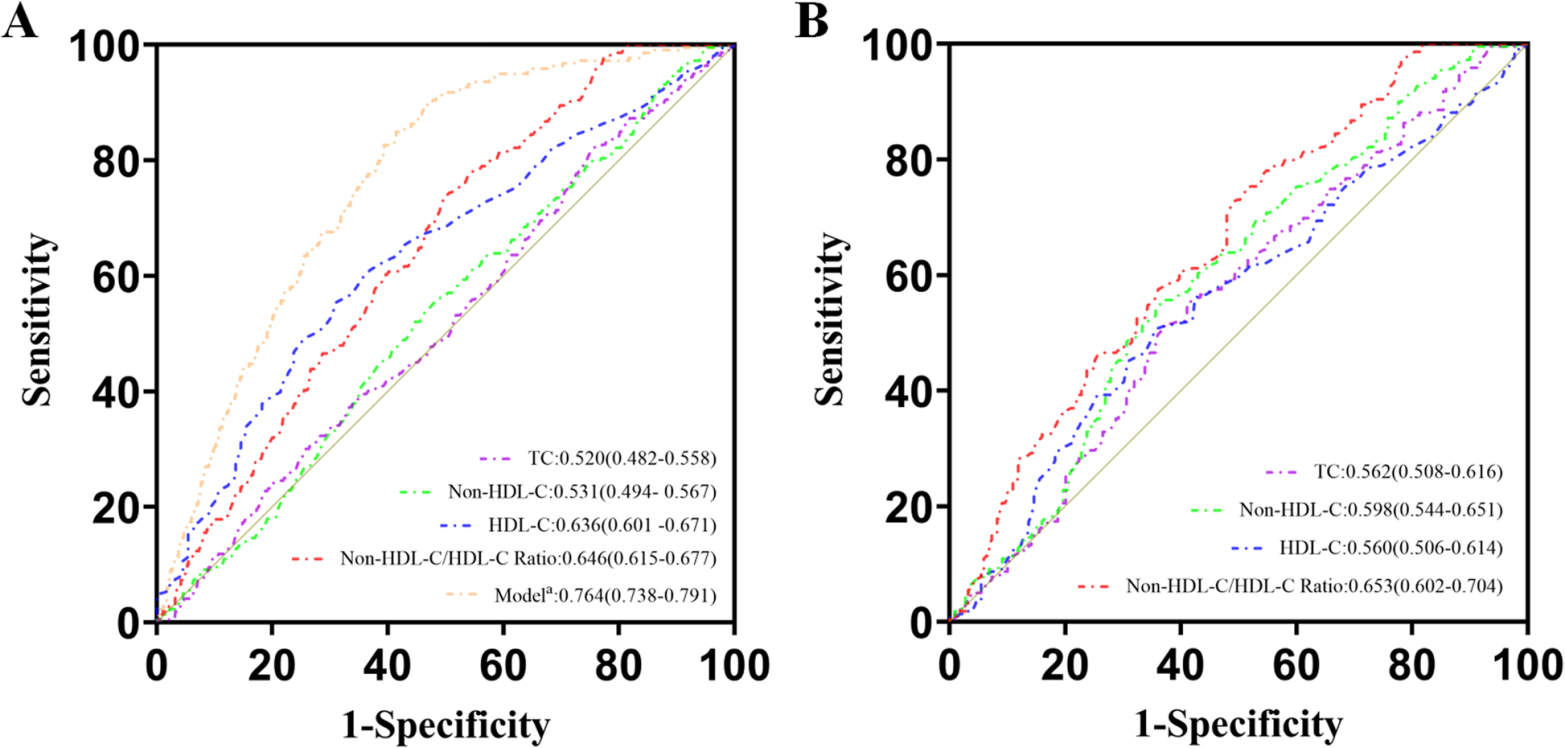
Receiver operating characteristic (ROC) curves of HDL-c, non-HDL-c and the non-HDL-c/HDL-c ratio for predicting the prevalence of AAA before (**A**) and after PSM (**B**). Model a was comprised of age, sex, smoking, hypertension, coronary artery disease and non-HDL-c/HDL-c ratio. HDL-c, high-density lipoprotein cholesterol; non-HDL-c, non-high-density lipoprotein cholesterol; non-HDL-c/HDL-c ratio, non-high-density lipoprotein cholesterol to high-density lipoprotein cholesterol ratio; PSM, propensity score matching

With the intention of calculating the predictive accuracy for NHHR, the ROC curve analysis was employed in this study. A comparison of HDL-C (AUC, 0.636; 95% CI, 0.601–0.671), non-HDL-C (AUC, 0.531; 95% CI, 0.494–0.567), TC (AUC, 0.520; 95% CI, 0.482–0.558) and NHHR (AUC, 0.646; 95% CI, 0.615–0.677) indicated that NHHR had the best predictive value. In addition, to enhance the diagnostic efficiency of NHHR, it was combined with the latest guideline-recommended risk determinants of AAA, which was comprised of age, gender, smoking and CAD, to form the Model a [35]. As a result, a favorable predictive performance was exhibited by the Model a (AUC, 0.764; 95% CI, 0.738–0.790) (Fig. 4A). Following PSM, ROC curve analysis was implemented, without certain confounding factors. Once more, the superior predictive value for AAA was demonstrated by NHHR (AUC, 0.653; 95% CI, 0.602–0.704) in comparison with non-HDL-C (AUC, 0.598; 95% CI, 0.544–0.651), TC (AUC, 0.562; 95% CI, 0.508–0.616) and HDL-C (AUC, 0.560; 95% CI, 0.506–0.614) as well (Fig. 4B). According to the YI, NHHR held the cutoff values, which were 2.83 before PSM and 2.75 after PSM. Based on these cutoff values, NHHR was divided into two groups [before PSM: low (< 2.83), high (> 2.83); after PSM: low (< 2.75), high (> 2.75)]. In contrast to the low NHHR group, both before and after PSM, the high NHHR group demonstrated a higher prevalence of AAA (Supplement Figure 1).

## Discussion

NHHR is a satisfactory diagnostic biomarker for AAA according to this study. As a result, AAA was found to be strongly associated with a high NHHR, which played a more important role than traditional lipid parameters in AAA screening among a Chinese population.

Among the numerous atherogenic lipid parameters presented, NHHR integrated all atherogenic cholesterols, including very low density lipoprotein cholesterol (VLDL-C), LDL-C, intermediate density lipoprotein cholesterol (IDL-C) and lipoprotein (a), in addition to HDL-C, which is an anti-atherogenic factor [24, 36, 37]. Lately, numerous researches has demonstrated a connection between NHHR and various dyslipidemia-related diseases, such as metabolic syndrome [18], liver disease [38], coronary atherosclerosis [39] and carotid atherosclerosis [20]. Moreover, dyslipidemia, especially the atherogenic dyslipidemia, affects the formation and progression of AAA [15, 40]. Iribarren and his colleagues considered that, when the levels of cholesterol exceed 240 mg/dl, it was in significant association with AAA (OR:2.82) [41]. As per the literature suggests,, an association was observed between the presence of AAA and HDL-C levels (MD, -0.15 mmol/L) [42]. Yasuhiko K et al. found that, in contrast to subjects in the lowest quintile of plasma lipoprotein(a), the individuals in the highest quintile exhibited a remarkable elevation on the subject of prevalence of AAA. (HR:1.57; 95% CI:1.19–2.08) through follow-up [43]. Nevertheless, there is a paucity of recent studies that have focused on the correlation between AAA and NHHR, which includes various atherogenic and antiatherogenic lipid particles. This study corroborated previous studies validated the correlation between HDL-C with AAA, as well as suggested NHHR could have a significant association with the prevalence of AAA.

However, the potential mechanism leading to NHHR induced prevalence of AAA was not fully expounded, and the damage of the atherogenic lipid particles to the aortic wall was only partly revealed. While it was documented that there is a notable association between AAA and atherosclerosis [15], it's an oversimplification to regard AAA merely as a upshot of advanced atherosclerosis [44]. The pathophysiological process is complicated and elusive and comprises three pivotal factors: proteolysis, smooth muscle cell apoptosis and inflammation [45]. A cohort study demonstrated a link between elevated LDL-C concentrations and matrix metalloproteinase-9 (MMP-9) allele [46]; in addition, the cholesterol metabolite, hydroxycholesterol (27-OHC), could increase MMP9 at the mRNA level [47]. Yin J et al. reported that cholesterol oxides might be able to trigger apoptosis in vascular smooth muscle cells based on animal experiments [48]. The intracellular redox system and activation of proinflammatory genes seemed to be changed by Lp(a), which led to the chronic inflammation in the aortic wall by means of its oxidized phospholipid content [49, 50]. Similarly, studies revealed that LDL-C could induce inflammation as well [51], and that modified LDL could lead to the NLRP3 inflammasome priming and activation in macrophages [52], of which affect formation of AAA [53]. Non-HDL-C, that is abundant and included more constituents than other lipoprotein particles, comprised all the morbific lipoproteins mentioned above. Apart from these effects, NHHR is adjusted by HDL-C, which exerts anti-inflammatory effects [54].

This study also verified that the association of NHHR with AAA existed in different age, sex, smoking, hypertension and CAD conditions, although are were all the risk factors of AAA [35]. The diagnostic value of NHHR is enhanced by its universality, especially in the widespread AAA screening. Owing to intact AAAs, which are commonly asymptomatic, an AAA screening program with ultrasonography demonstrated timely diagnosis of AAAs and reduced AAA-related mortality [55]. Although the US Preventive Services Task Force have advocated a single AAA ultrasound screening for male individuals between 65 and 75 years old that have a smoking history [56], AAA screening is prone to trigger overdiagnosis. Therefore, NHHR is anticipated to assist in identifying high-risk AAA individuals, while improving screening diagnostic accuracy, thus preventing overdiagnosis during AAA ultrasound screening.

### Strengths and limitations

There are a few limitations to be acknowledged in the current research. First, due to its cross-sectional nature, this study might be affected by selection bias.. However, to minimize potential bias in the study, both PSM and multivariable logistic regression analyses were employed. Additionally, this study could not establish causative links. Second, only once was the lipid profile evaluated and noted. A lack of reduplicated measurement of the lipid profile could lead to the influence of acute stress and occasionality. Third, although we have already supplemented some of the patient's medication information, there was no detailed information about previous use of drugs, including specific lipid-lowering medications, dosing frequency, and duration of medication use. Therefore the influence of drugs, such as stains, could not be adjusted accurately. Finally, this study was only consisted of 219 AAA patients. However, this was a persistent study with durative AAA screening in the hospital and communities. In prospective research, we intend to prioritize exploring the diagnostic significance and prognostic assessment of NHHR.

## Conclusion

In addition to the traditionally pivotal lipid parameters, NHHR was in association with AAA independently. The association existed in different age, sex, smoking, hypertension and coronary arterial disease conditions. Clinicians could utilize NHHR to assist in identifying high-risk AAA individuals, and improve efficiency of screening, thus preventing overdiagnosis during AAA ultrasound screening.

### Supplementary Information

**Additional file 1: Supplementary Figure 1.**

**Additional file 2: Supplementary Figure 2.**

**Additional file 3: Supplementary Figure 3.**

**Additional file 4: Supplementary Table 1.** Baseline characteristics of all the participants.

**Additional file 5: Supplementary Table 2.** Multicollinearity test of variable in multivariable logistic regression analysis.

## Data Availability

Provided there is a valid and reasonable request made for the information derived from this research, it can be obtained from the primary or corresponding author.

## Abbreviations

AAA: Abdominal aortic aneurysms
CAD: Coronary heart disease
Non-HDL-C: Non-high-density lipoprotein cholesterol
AUROC: Areas under the receiver operating characteristic curves
Non-HDL-C/HDL-C: Non-high-density lipoprotein cholesterol to high-density lipoprotein cholesterol
NHHR: Non-high-density lipoprotein cholesterol to high-density lipoprotein cholesterol ratio
TC: Total cholesterol
AAD: Abdominal aortic diameter
LDL-C: Low-density lipoprotein cholesterol
SMD: Standardized mean differences
IQR: Interquartile range
ORs: Odds ratios
CIs: Confidence intervals
BUN: Blood urea nitrogen
PSM: Propensity score matching
ROC: Receiver operating characteristic
AUC: Area under the curve
VLDL-C: Very low density lipoprotein cholesterol
IDL-C: Intermediate density lipoprotein cholesterol
MMP-9: Matrix metalloproteinase-9
