# Supplementary figures and images for: Association between the non-HDL-cholesterol to HDL- cholesterol ratio and abdominal aortic aneurysm from a Chinese screening program
Source: Lipids Health Dis. 2023 Nov 6;22:187. doi: 10.1186/s12944-023-01939-4 (PMC10626699; doi:10.1186/s12944-023-01939-4)

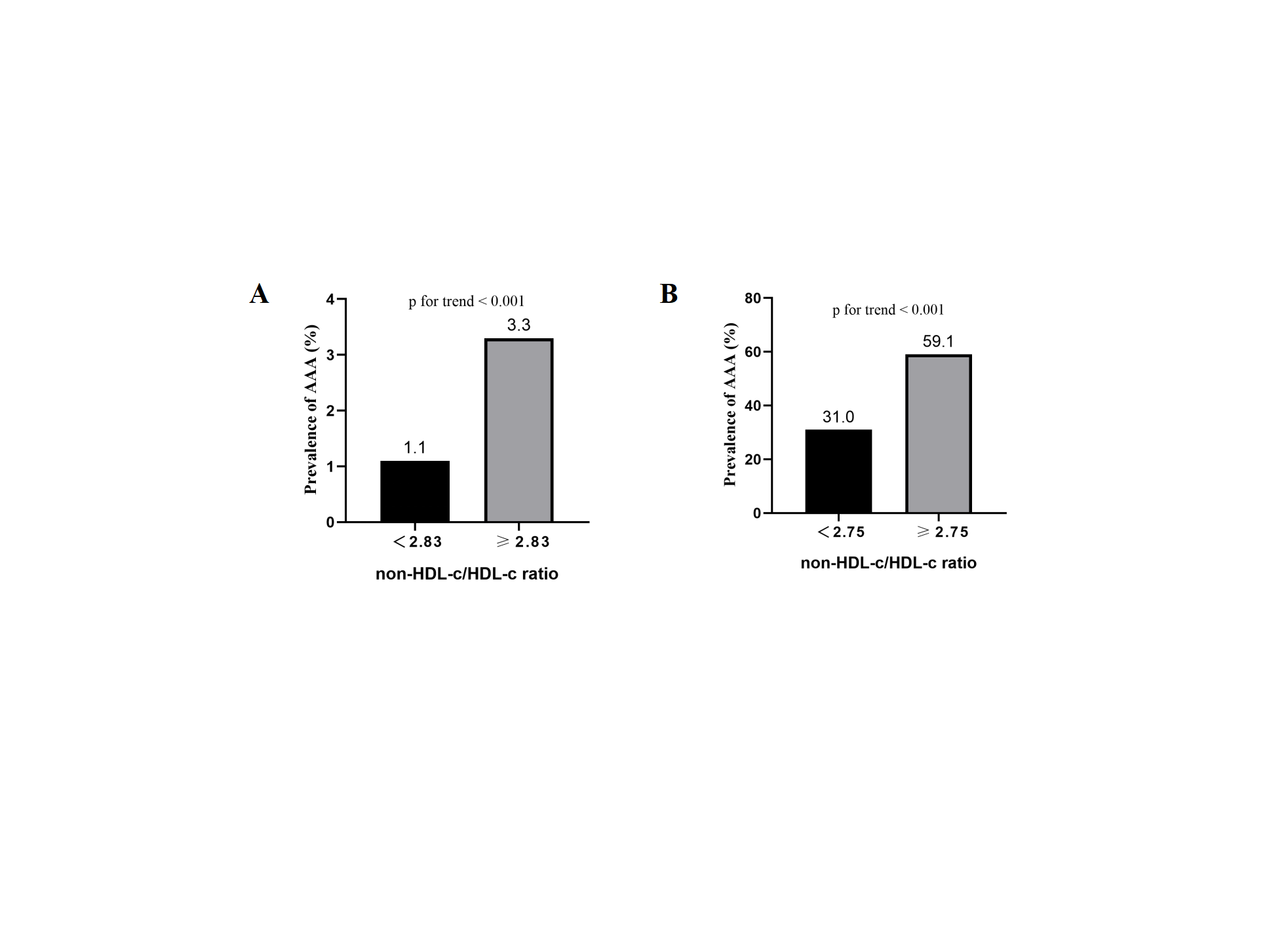

Supplement: Supplementary file 1 — Additional file 1: Supplementary Figure 1. [file 12944_2023_1939_MOESM1_ESM.tif]
