# Supplementary material for: Association between the non-HDL-cholesterol to HDL- cholesterol ratio and abdominal aortic aneurysm from a Chinese screening program
Source: Lipids Health Dis. 2023 Nov 6;22:187. doi: 10.1186/s12944-023-01939-4 (PMC10626699; doi:10.1186/s12944-023-01939-4)
Supplement: Supplementary file 3 — Additional file 3: Supplementary Figure 3. [file 12944_2023_1939_MOESM3_ESM.pdf]

SPRINGER NATURE
Author Services

Editing Certificate

This document certifies that the manuscript

Association between the non-HDL-cholesterol to HDL- cholesterol ratio with Abdominal Aortic Aneurysm from a Chinese Screening program

prepared by the authors

Wenhui Lin, Songyuan Luo, Wei Li, Jitao Liu, Ting Zhou, Fan Yang, Dan Zhou, Yuan Liu, Wenhui Huang, Yingqing Feng, Jianfang Luo

was edited for proper English language, grammar, punctuation, spelling, and overall style by one or more of the highly qualified native English speaking editors at SNAS.

This certificate was issued on **September 22, 2023** and may be verified on the SNAS website using the verification code **DB60-000C-D525-38C8-CB69** .

Neither the research content nor the authors' intentions were altered in any way during the editing process. Documents receiving this certification should be English-ready for publication; however, the author has the ability to accept or reject our suggestions and changes. To verify the final SNAS edited version, please visit our verification page at secure.authorservices.springernature.com/certificate/verify.
If you have any questions or concerns about this edited document, please contact SNAS at support@as.springernature.com.

SNAS provides a range of editing, translation, and manuscript services for researchers and publishers around the world.
For more information about our company, services, and partner discounts, please visit authorservices.springernature.com.
