# Supplementary material for: Association between the non-HDL-cholesterol to HDL- cholesterol ratio and abdominal aortic aneurysm from a Chinese screening program
Source: Lipids Health Dis. 2023 Nov 6;22:187. doi: 10.1186/s12944-023-01939-4 (PMC10626699; doi:10.1186/s12944-023-01939-4)
Supplement: Supplementary file 4 — Additional file 4: Supplementary Table 1. Baseline characteristics of all the participants. [file 12944_2023_1939_MOESM4_ESM.docx]

**Supplementary TableⅠ.Baseline Characteristics of all the participants.^a^**

| Variables | Total (n=9559) |
| --- | --- |
| Age (years) | 70.3±0.1 |
| BMI (Kg/m^2^) | 23.9±0.03 |
| Sex (male) | 64.3 (6144) |
| Smoking, % | 24.1 (2304) |
| Abdominal aortic aneurysm, % | 219 (2.3) |
| Hypertension, % | 54.3 (5193） |
| Diabetes mellitus, % | 21.5 (2054） |
| Coronary artery disease, % | 39.2 (3750） |
| Peripheral artery disease, % | 3.3 (320） |
| Stroke, % | 5.5 (525) |
| Maximal abdominal aortic diameter (mm) | 19.0 (17.0-20.0) |
| ALT (U/L) | 20.0 (14.6-27.6) |
| AST (U/L) | 22.0 (18.3-28.0) |
| UA (mmol/L) | 399.0 (328.0-461.0) |
| CR (μmol/L) | 81.3 (67.2-97.0) |
| BUN (mmol/L) | 5.5 (4.5-6.9) |
| TG (mmol/L) | 1.4 (1.0-1.9) |
| TC (mmol/L) | 4.6 (3.8-5.4) |
| LDL- C (mmol/L) | 2.8 (2.2-3.5) |
| HDL-C (mmol/L) | 1.1 (0.9-1.4) |
| non-HDL-C (mmol/L) | 3.4 (2.7-4.2) |
| non-HDL-C / HDL-C ratio | 3.0 (2.2-3.9) |
| Fasting glucose (mmol/L) | 5.3 (4.7-6.3) |
| HBA1C (mmol/L) | 6.1 (5.7-6.3) |
| Medication use | |
| Angiotensin system inhibitors, % | 47.7 (4563) |
| Beta-blockers, % | 42.6 (4069) |
| Statins, % | 51.4 (4909) |
| Metformin, % | 9.5 (912) |

Abbreviations: AAA, abdominal aortic aneurysm; BMI, body mass index; ALT, alanine aminotransferase; AST, aspartate aminotransferase; UA, uric acide; Cr, creatinine; BUN, blood urea nitrogen; TG, triglyceride; TC, total cholesterol; LDL-C, low-density lipoprotein cholesterol; HDL-C, high-density lipoprotein cholesterol; non-HDL-C, non-high-density lipoprotein cholesterol; non-HDL-C/HDL-C ratio, non-high-density lipoprotein cholesterol to high-density lipoprotein cholesterol ratio; HbA1c, hemoglobin A1c; SMD, standardized mean difference.

^a^ Values are given as mean + standard deviation, number (percentage), or median (quartiles 1 through 3).
