# Supplementary material for: Association between the non-HDL-cholesterol to HDL- cholesterol ratio and abdominal aortic aneurysm from a Chinese screening program
Source: Lipids Health Dis. 2023 Nov 6;22:187. doi: 10.1186/s12944-023-01939-4 (PMC10626699; doi:10.1186/s12944-023-01939-4)
Supplement: Supplementary file 5 — Additional file 5: Supplementary Table 2. Multicollinearity test of variable in multivariable logistic regression analysis. [file 12944_2023_1939_MOESM5_ESM.docx]

**Supplement. Table Ⅱ. Multicollinearity test of variable in multivariable logistic regression analysis.**

|  | Unmatched population | |  |  | Matched population | |
| --- | --- | --- | --- | --- | --- | --- |
| Variables | Tolerance | VIF |  | Variables | Tolerance | VIF |
| Age | 0.901 | 1.110 |  | ALT | 0.976 | 1.024 |
| Sex | 0.770 | 1.299 |  | UA | 0.955 | 1.047 |
| Smoking | 0.852 | 1.174 |  | HDL-c | 0.549 | 1.820 |
| Hypertension | 0.764 | 1.308 |  | LDL-c | 0.495 | 2.022 |
| Coronary artery disease | 0.529 | 1.890 |  | Non-HDL-c/HDL-c ratio | 0.441 | 2.270 |
| Stroke | 0.974 | 1.026 |  |  |  |  |
| UA | 0.809 | 1.236 |  |  |  |  |
| Cr | 0.537 | 1.861 |  |  |  |  |
| BUN | 0.503 | 1.987 |  |  |  |  |
| Non-HDL-c/HDL-c ratio | 0.630 | 1.586 |  |  |  |  |
| HDL-c | 0.538 | 1.859 |  |  |  |  |
| HBA1C | 0.953 | 1.049 |  |  |  |  |
| Angiotensin system inhibitors | 0.662 | 1.512 |  |  |  |  |
| Beta-blockers | 0.546 | 1.832 |  |  |  |  |
| Statins | 0.449 | 2.226 |  |  |  |  |
